# Personal

**Published:** 1895-07

**Authors:** 


### Personal.

DR. JOHN B. MURPHY, of Chicago, whose intestinal anastamosis button has so justly made him famous, recently has been elected president of the national association of railway surgeons. The *International Journal of Surgery* very gracefully says that, "of the available candidates, no better selection could have been made. Dr. Murphy's election will go far in restoring harmony. It will strengthen the old and attract new blood to the body which has placed him at its head. Dr. Murphy will preside over the various sessions of the national association with his usual dignity, grace and ability."

We cordially agree with our contemporary in its opinion of the distinguished Chicagoan.

---

DR. HORACE CLARK, of Buffalo, announces that he will have office hours at 21 North street from 1 to 4 P. M.; also at the Morgan building, Niagara and Pearl streets, room 210, from 10 A. M. to 12.30 P. M., and 4.30 to 5.30 P. M., except Sundays. His practice is limited to diseases of the nose, throat and chest.

Dr. Herman Mynter, of Buffalo, sailed Saturday, June 8, 1895, in the steamer "Hekla," Thingvalla line, with his family, for a summer visit to his former home, Copenhagen. He will return early in August to resume his surgical practice.

---

Dr. John E. Bacon has located at 149 Franklin street, Buffalo. Hours, 8–11 A. M., 4–6 P. M. Telephone, Seneca 509.

---

Dr. William C. Fritz, of Buffalo, has removed his office from 235 East North street to 164 East North street.

---

Dr. Walter D. Greene, of Buffalo, has removed from 365 North Division street to 385 Jersey street.

---

Dr. Thomas B. Carpenter, of Buffalo, has removed from 660 Main street to 325 Jersey street.

---

Dr. Irving M. Snow has been appointed associate editor of the *Archives of Pediatrics.*

